# The Mammary Gland Carcinogens: The Role of Metal Compounds and Organic Solvents

**DOI:** 10.1155/2013/640851

**Published:** 2013-05-15

**Authors:** Stephen Juma Mulware

**Affiliations:** Ion Beam Modification and Analysis Laboratory, Physics Department, University of North Texas, 1155 Union Circle, #311427, Denton, TX 76203, USA

## Abstract

The increased rate of breast cancer incidences especially among postmenopausal women has been reported in recent decades. Despite the fact that women who inherited mutations in the BRCA1 and BRCA2 genes have a high risk of developing breast cancer, studies have also shown that significant exposure to certain metal compounds and organic solvents also increases the risks of mammary gland carcinogenesis. While physiological properties govern the uptake, intracellular distribution, and binding of metal compounds, their interaction with proteins seems to be the most relevant process for metal carcinogenicity than biding to DNA. The four most predominant mechanisms for metal carcinogenicity include (1) interference with cellular redox regulation and induction of oxidative stress, (2) inhibition of major DNA repair, (3) deregulation of cell proliferation, and (4) epigenetic inactivation of genes by DNA hypermethylation. On the other hand, most organic solvents are highly lipophilic and are biotransformed mainly in the liver and the kidney through a series of oxidative and reductive reactions, some of which result in bioactivation. The breast physiology, notably the parenchyma, is embedded in a fat depot capable of storing lipophilic xenobiotics. This paper reviews the role of metal compounds and organic solvents in breast cancer development.

## 1. Introduction

Breast cancer accounts for 16% of all cancer deaths among women globally, according to the report by the World Health Organization [1]. It is the most common solid tumor diagnosed in women [2] and there is also an increasing trend in men. Even though some women are genetically predisposed by inheriting the BRCA1 and BRCA2 genes, studies show that an elevated lifetime of estrogen exposure is also another risk factor for breast cancer [3]. The ability of estrogen and estrogen metabolites to generate oxygen reaction species (ROS) which can induce DNA synthesis, increase phosphorylation of protein kinases, and activate transcription factors like AP-1, NRF1, E2F, and CREB which are found to be responsive to oxidants (including solvent toxins and metal compounds) embodies the basis of carcinogenesis by estrogen [4]. Whereas the activation of transcription factors plays a vital role in cell transformation, cell cycle, migration, and invasion, and it increases the genetic instability [5]. 

 Occupational and environmental exposure to metal compounds and organic solvents have contributed to increasing breast cancer cases in the recent decades especially as many women have entered work places since 1960s [6]. It has been reported that metallic compounds could function as estrogen disruptors [7] as well as carcinogenic agents. Generally, metal genotoxicity is caused by indirect mechanism including interference with cellular redox regulation and induction of oxidative stress, inhibition of DNA repair system, and deregulation of cell proliferation by induction of signaling pathways or inactivation of tumor suppressor genes. In addition, the increased DNA methylation especially in an invasive or tumor stage has been reported widely as a leading factor in breast carcinogenesis by various studies using RLGS. For instance, studies of human breast tissues determined that NRG1 exon 1 (neuregulin 1) was partially methylated in 14 out of 17 (82.4%) of the invasive carcinomas samples tested, while it was unmethylated in 8 out of 10 of the normal breast tissues. Partial methylation was also found in 9 out of 14 (64.3%) morphologically normal tissue samples adjacent to invasive carcinomas sites [8]. Another study reported that DNA methylation exhibits various patterns in different cancer types since methylation is most significantly linked with radical changes in conditional concordant relationships (CCRs) throughout the genome. Hypermethylation of DNA thus enhances progression, cell migration, and oncogenicity in breast cancer [9]. Some metals also undergo metabolic transformation such as reduction to lower oxidation state or alkylation [10]. A unique feature of metal biology is the fact that even essential metals like iron and copper can become toxic depending on the oxidation state, complex form, dose, and mode of exposure.

Organic solvents are widely and routinely used domestically as part of consumer products or in various industries, and they can get into the body through inhalation, ingestion, or by dermal exposure. While exposure is the highest in some work places, most of these solvents and other chemicals are commonly found in ambient air, drinking water, and consumer products [11, 12]. The detection of some solvents in the some human breast milk confirms that after exposure, these compounds can find their way into the breast tissues [13]. Organic solvents and their metabolites are believed to initiate or promote breast cancer through genotoxic or other related mechanisms including mutagen or tumorigen among others. Generally, the basis of organic solvent carcinogenesis depends on the estrogenic properties of halogenated hydrocarbons which are implicated in the etiology of breast cancer. Similarly, another hypothesis is based on the fact that the lipophilic substances and their metabolites can migrate to the adipose tissue in the breasts where they can be biotransformed in situ and then excreted into ductular systems where while in contact with breast parenchyma for long enough can initiate or promote carcinogenesis through genotoxic or related mechanism [14]. Several epidemiological studies in the recent past have established that organic solvents play a role in breast carcinogenesis. For instance, a well-designed epidemiological study among young Danish women in occupations with greater exposure of solvents found elevated risks of breast cancer [15]. This study established that the risk was doubled among women who had over 10 years of exposure. Another case-registry-based study of Canadian women established elevated risks among pre- and postmenopausal women who were employed in two industries with high chemical exposure. Those in a dry cleaning work place which used tetrachloroethylene recorded even higher rates of breast cancer [16]. This paper will discuss breast physiology as an enabling factor especially for organic solvents, absorption and distribution of carcinogens, physiochemical properties, and the general metal genotoxicity mechanisms. It will then highlight specific metal compounds and organic solvents that have been linked to breast cancer. 

## 2. Breast Parenchyma

In anatomical terms, the parenchyma of a body part constitutes all the cells of that organ that have an essential function. Breast parenchyma is made of a series of apocrine glands which includes the milk ducts and the glands that produce the milk. These epithelial cells which are arranged into lobules connected to ductular system of the breast end up at the nipple. The breast parenchyma are embedded in fatty tissue, the amount of which varies from one woman to another, and connective tissues with rich supply of blood and lymphatic vessels. The breast physiology comprises certain unique features that make it sensitive to various metal compounds and organic solvents and their metabolites that may expose it to cancer development. First, the apocrine glands that make up the breast parenchyma proliferate continuously from the menarche with increasing cell population during every ovulatory cycle of a female resulting in the development of the budding structure until approximately the age of 35 [14]. A mutation occurring during DNA transcription and other errors that may occur during such replication expose breast tissues to high risk. Furthermore, the accumulation of xenobiotics in the breast tissues is enhanced by physiological changes that take place in the body during menstrual cycles. 

Due to reduction in the metabolic and clearance activity of the breast tissue, the byproducts of metabolic oxidative and reductive processes (redox) remain in the breast parenchyma for too long to act as early stage initiators, through changes in DNA bases and other hydroxyl radical-induced adverse reactions [17]. They can also act as promoters of already initiated cells, with both theories consistent with the observation of nearly 70% of breast tumors originating in the ductular system [18]. Exogenous carcinogens including metal compounds and organic solvents are secreted into breast fluids as evidenced by a unique study among boat women of Aberdeen in Hong Kong. The study found that these women who traditionally nursed their infants only from their right breast and not their left breast were as twice as likely to develop breast cancer on the left breast due to stagnation of the milk there, with the risk increasing more with age [19]. In a separate study, breast secretions and colostrums especially among farm women who were exposed to more carcinogens were found to be mutagenic on an Ames test [20].  Table 1 shows organic solvents that have been detected in human milk. 

## 3. General Mechanism of Metal Carcinogenicity

Metals can be carcinogenic in various forms including free ions, metal complexes, or particles as well as soluble metal compounds. The physiochemical properties of various metals govern their respective toxicity. Oxidation state, charge, and ionic radii play significant role with regards to metal free ions while the coordination number, the geometry, and the type of legand (i.e., their lipophilicity) determine their toxicity. On the other hand, the soluble metal compounds' toxicity depends in part on the particle size and crystal structures [10]. If toxic metal ions have similar physiochemical properties like charge and size compared to essential metal ions, they tend to compete for biological binding sites of the latter resulting into structural perturbations leading to aberrant function of biological macromolecules [21, 22]. In general, metal carcinogenicity and genotoxicity are based on three main mechanisms, namely, oxidative stress, DNA repair modulation, and disturbances of signal transduction pathways. 

### 3.1. Induction of Oxidative Stress

Ions of carcinogenic metals like arsenic, antimony, chromium, lead, cobalt, nickel, and vanadium produce redox reactions in biological systems. They induce the formation of reactive oxygen and nitrogen species in mammalian cells. The oxidative concept in metal carcinogenesis signifies that the complexes formed by these metals, in vivo, in the vicinity of the DNA, catalyze redox reactions oxidizing DNA in turn [26]. The most significant effect of reactive oxygen species (ROS) in the carcinogenic progression is the DNA damage, which in turn results into lesion-like strand breaks and the sister-chromatid exchange [27]. Redox processes lead to the formation of hydroxyl radicals by Fenton and Haber-Weiss reactions which in turn cause oxidative damage to lipids proteins and DNA. There is a fine balance between enzymatic (such as super oxide dismutase, SOD, glutathione peroxidase, and catalase) and nonenzymatic (like ascorbic acid, *α*-tocopherol, and *β*-carotene and isoflavones) antioxidants and free radicals in each cell. A higher ROS generation can also lead to lipid peroxidation, depletion of the sulfhydryl groups, and change of transduction pathways leading to cell apoptosis. Although cadmium ions do not exert redox reactions in biological systems, they still generate oxidative stress by inhibiting antioxidative enzymes in vivo and in vitro. 

Iron and copper are also effective catalysts for fenton and fenton-type reactions. The two metals are, however, heavily regulated with respect to uptake, transport, storage, mobilization transfer to target molecules, and excretion thus ensuring that the increased deliberation of free ions is restricted to conditions of extreme overload, genetic defects in metal homeostasis, and/or metabolic stress [10]. Copper plays a role in activation of more than 30 enzymes including among others ceruloplasmin, cytochrome oxidase, lysine oxidase, dopamine hydroxylase, and ascorbate oxidase, some of which are involved in the synthesis of the main component of connective tissues called collagen. Iron also plays a vital role in oxygen transportation, xenobiotic metabolism, and oxidative phosphorylation and acts as prosthetic group in many enzymes [28]. 

### 3.2. DNA Repair Inhibition

The process of DNA repair in mammalian cells is a multipathway mechanism meant to protect cells from the plethora of DNA damaging agents known to attack nuclear DNA. Most of anticancer therapies including ionizing radiation and chemotoxic therapies rely on this capability to create DNA lesions, leading to apoptosis/cell death [29]. Most carcinogenic metals usually are comutagenic, which means that they enhance the mutagenicity of other genotoxic agents. At low concentrations, most of these metals inhibit repair of DNA damage caused by either xenobiotics or endogenous factors [30]. Inhibition of DNA repair mechanisms, including nucleotide excision repair, base excision repair, DNA-SSB rejoining, and *O* ^6^-methylguanine-DNA methyltransferase [31–33] is of high relevance for human carcinogenesis, since DNA repair inhibition by cadmium, for instance, occurs at very low concentrations even though most direct DNA damaging effects are usually observed at higher concentrations. Inhibition of DNA repair may also explain the observation that cadmium, despite being nonmutagenic in bacteria, enhances mutagenicity of methyl nitrosourea and strongly enhances UV-induced mutagenicity in mammalian cells as well [34]. DNA repair mechanisms are frequent targets for interference by carcinogenic metal compounds. Inhibition of repair and continuous DNA damage results in genomic instability, which under conditions of accelerated cell proliferation and impaired apoptosis may become deleterious. 

### 3.3. Deregulation of Cell Proliferation

Deregulation of cell growth and differentiation are two main factors that characterize tumor development. Carcinogenic metals can alter mammalian cell growth by affecting the expression of growth stimulating factors through activating mitogenic signaling pathways and inducing the expression of cellular protooncogens. At the same time, modified gene expression can result from epigenetic mechanism like hypo- or hypermethylation of DNA and/or disturbed histone acetylation. The other way the carcinogenic metals can alter mammalian cell growth is by inactivating growth control mechanism [10]. In general, the deregulation of cell proliferation that leads to tumor growth and invasive cancer stage is often imposed by viral agents, heavy metals, toxins, and oxidants. Some metal carcinogens have been shown to inactivate the tumor suppressor proteins p53. Metal ions can also deregulate cell proliferation by inactivating apoptotic process resulting in adaptation to the cytotoxicity of the metal.

## 4. Specific Metal Mammary Gland Carcinogens

### 4.1. Arsenic

Arsenic is commercially produced as a byproduct of nonferrous metal production, precisely from copper smelting. Arsenic exposure constitutes one of the most wide spread environmental carcinogens associated with numerous cancers. Arsenites are found in drinking water, some wood preservatives, insecticides, and herbicides. In the natural waters, arsenic occurs in oxidation states III and V in the form of arsenous acid (H_3_AsO_3_) and arsenic acid (H_3_AsO_5_) and their salts [35]; at the same time smaller amounts of monomethylarsonic acid (MMA) and dimethylarsinic acid (DMA) can also be present. The trivalent monomethylated (MMAIII) and dimethylated (DMAIII) arsenic species have been detected in lake water [36]. The International Agency of Research on Cancer (IARC) in its evaluations classified arsenic and its compounds as highly carcinogenic to humans [37]. Among the cancers linked to arsenic compounds are skin, bladder, liver, kidney, lung and breast cancers [38]. 

The main source of exposure to humans is through drinking ground water with high level of arsenic contamination especially in South Asia region. This region witnessed wide spread carcinogenic effects from the contaminated water that led USEPA to declare the maximum contaminant limit to 0.010 mg/L in line with WHO guidelines [36]. The rice planted by irrigation in these regions has also, through uptake, introduced arsenic into the human food. Among other major exposure mechanisms of arsenic to human food is through consumption of contaminated fish and sea foods. These exposure mechanisms can be easily reduced through effective food evaluation by authorities and enhanced education to the populations in the regions affected, alongside minimizing industrial exposures. 

Various methods have been developed and improved for the measurement of total arsenic exposure agents especially foods and drinking water and have been widely used for the evaluation of the quantitative or qualitative level of contamination and the resulting concentrations of arsenic in humans. Similarly, analytical methods allowing arsenic speciation have attracted more research in light of its carcinogenic potential. The form of existence of the arsenic compounds dictates its environmental fate and behavior, bioavailability, and toxicity, for instance, the inorganic AsIII and AsV are far more toxic than MMA and DMA [36].

Arsenic trioxide (As_2_O_3_) which has been used as a component of Chinese medicine for hematologic malignancy treatment induces cell cycle arrest and apoptosis in solid tumors including breast cancer [39]. It is hoped that new insights into how the As_2_O_3_ binds to specific receptors and how they trigger signaling pathways may help explain its anticancer strategies that may be helpful in the treatment of other solid tumors including breast cancer. High environmental concentration and thus exposure of arsenite (5 *μ*M/0.65 mg/L) can induce both replications-dependent DNA double-strand breaks and homologous recombination. The study found that the double-strand break was replication dependent which might have resulted from conversion of a DNA single-strand break to double-strand break [40]. On the other hand, low arsenite concentration induces ROS production and ROS depolarization of the mitochondria membrane. When the ROS mediated DNA damage was measured by the presence of 8-OHdG DNA adducts in the nuclei, I*κ*B phosphorylation, NF-*κ*B activation, and increase in c-Myc and HO-1 protein levels were observed. The role of arsenic induced breast cancer cell, MCF-7, recruitment into the S-phase of cell cycle and cell proliferation can be explained by the above factors. The study, however, concluded that arsenates activates many pathways involved in MCF-7 cell proliferation leading to a suggestion that arsenite exposure may be a high risk cause to breast cancer [38]. Accumulating evidence from cell culture studies and from arsenic exposed humans suggests that arsenic changes the DNA methylation pattern, hence affecting the expression of oncogenes and tumor suppressor genes. 

### 4.2. Cadmium

Cadmium is emitted to the air by mines, metal smelters, and industries which use cadmium compounds for in the production of alloys, batteries, pigments, and plastics [30]. It is a widespread environmental pollutant that has been declared carcinogenic to humans by the International Agency of Cancer Research [37]. Both active and passive smoking of all forms of tobacco is the single most means of exposure to humans, since all forms of tobacco contain high levels of cadmium. This is so since the absorption of cadmium in tobacco smoke from the lungs into the body cells is much greater than the gastrointestinal tract absorption [41, 42]. It is important to note that tobacco also contains other 16 carcinogenic compounds apart from cadmium, including benzo[*a*]pyrene (BaP), 4-(methylnitrosamino)-1-(3-pyridyl)-1-butanone (NNK) and *N*′-nitrosonornicotine (NNN), 2-naphthylamine, 4-aminobiphenyl, formaldehyde, 1,3-butadiene, benzene, vinyl chloride, ethylene oxide, arsenic, beryllium, nickel compounds, chromium VI, and polonium-210 which have been classified by IARC as group 1 carcinogens [25]. Since cadmium ions can easily be absorbed and stored in plants roots, stems, leaves and fruits, consumption of such affected parts of plants by animals may lead to contamination of their milk and meat products, which in effect can contaminate human food [42]. Sea foods such as mollusks and crustaceans have also been a source of contamination [43, 44]. 

Cadmium is a ubiquitous carcinogenic pollutant as already mentioned, with multiple biological effects on living cells and various case studies have correlated its exposure to breast cancer [2, 29, 45, 46]. These studies observed a significant increased risk of breast cancer in patients with higher urinary cadmium concentration. Cadmium is believed to have diverse effects in various cellular processes including cell proliferation, differentiation, and apoptosis. It is an endocrine disruptor that stimulates estrogen-receptor *α* (ER-*α*) activity promoting uterine and mammary gland growth while at the same time impairing the cancer protecting effect of selenium in female inbred C3H mice carrying murine mammary tumor virus [47]. Cadmium changes intercellular signals by modulating gene expression and affecting the patterns of transcriptional activity. It activates extracellular regulated kinases erk-1 and -2, in both ER-positive and ER-negative human breast cancer cells while high cadmium concentrations induce a proliferative response SKBR3 cells and increase intracellular cAMP levels [48]. Cadmium also increases breast cancer proliferation in vitro by stimulating Akt, ERK1/2, and PDGFR*α* kinases activities most likely by activating c-fos, c-jun, and PDGFA by an ER-*α*-dependent mechanism [49]. Another study also found that cadmium despite being able to promote carcinogenesis can also delay the onset of tumors by inhibiting breast cancer cell-induced angiogenesis [50]. 

### 4.3. Lead

Lead exists both as free metal and also in various compounds. It can also be found in plants, animals, air, water, dust, and soil. Inorganic lead compounds like tetraethyl lead and tetramethyl lead are combined with carbon groups while lead oxide and lead chloride are combinations of lead with other compounds. The main exposure mode to human is through inhalation from dust or fumes and by swallowing. Most observed mechanisms of lead carcinogenicity involve direct DNA damage, oxidative DNA damage through ROS generation, clastogenicity, or inhibition of DNA synthesis or repair [51, 52]. Lead is also known to substitute for zinc in several proteins that function as transcriptional regulators hence reducing the binding of these proteins to the recognition elements in the genomic DNA. This process leads to a suggestion that lead is involved in epigenetic alteration of gene expression [53].

In animal experiments of female C3H mice, low concentration of lead in drinking water was found to promote the development and accelerate the growth of mammary murine tumors [3]. High levels of lead were found in blood and hair of newly diagnosed patients of breast cancer by the same study, with the lead levels in the hair directly correlated to the volumes of the tumors present. The same study also established that lead interacted with iodine, an essential trace element that most likely protects against breast cancer, by substituting it in the cell tissues. Lead-zinc interactions in proteins can also cause posttranslational changes in the protein structure. This occurs when lead displaces zinc in a tumor suppressor protein p53 resulting in a structurally altered form with functional mutation consequences involving deletion of p53 as observed with cadmium [54]. As one molecular target with respect to base excision repair, lead inhibits the apurinic/apyrimidinic endonuclease (APE1) in the low micromolar concentration range both in an isolate enzymic test system and in cultured AA8 cells. This leads to an accumulation of apurinic sites in DNA and increase in MMS-induced mutagenicity [55]. Lead also interferes with the repair of DNA double strand breaks through interaction with the stress response pathway induced by ATM (a phosphoinositide-3-kinase) [56].

### 4.4. Nickel

Most nickel on earth is mainly found in the iron-nickel molten ore, which constitutes 10% of nickel. Coal and oil contains considerable amounts of nickel since it is readily absorbed in organic matter. Human exposure occurs through contaminated food and drinking water, smoking cigarettes, and contact with nickel contaminated soil or water. Occupational exposure is common in industries that handle nickel laced materials like high temperature oxidation of nickel matte and nickel-copper matte, electrolytic refiners, and during nickel salt extraction in hydrometallurgical industry. There has been reported experimental evidence that nickel metal dust can become solubilized and bioavailable after inhalation [57].

 Nickel compounds are known carcinogens in both human and animal models [58]. The genotoxic effects of nickel compounds may be indirect through the inhibition of DNA repair systems [59, 60] while nickel carcinogenesis is believed to be mediated by oxidative stress, DNA damage, epigenetic effects, and the regulation of gene expression by activation of certain transcription factors [61]. Nickel has been shown to stimulate cell growth in estrogen receptor (ER) positive breast cancer cells, MCF-7 [62]. Another study by Ionescu et al. [63] established highly significant nickel accumulation in 20 breast cancer tissue biopsies compared to controls.

### 4.5. Organic Solvents

The toxicity of organic solvents in the human body is governed by the extent of absorption, distribution, and metabolization. Once they enter the body through ingestion, inhalation, or cutaneous absorption, they enter the blood stream through which they are transported to various organs [63]. Most blood rich organs including the lungs, kidney, and liver take up much of the organic solvents while the rest are distributed and absorbed by fatty tissues in other organs. Once stored in the surrounding fatty tissues of the breast, the organic solvents migrate into the lobules and the ductular system as secretory products of the epithelial cells, and by simple, passive, or active diffusion through cell membranes, by direct transport through water-filled pores in the cell membranes [64]. 

## 5. Specific Organic Solvent Mammary Gland Carcinogens

### 5.1. Benzene

Benzene is a volatile organic compound widely used in various chemical production including gasoline production, storage, transport, vending, and combustion. It is also a byproduct of processes like coke oven as well as being a component of various consumer products like carpets, pesticides, and adhesive removers [65]. Benzene is both air and water contaminant closely monitored by most states in the USA. Apart from air pollution resulting from industrial burning, exhaust, and gas fumes, as well as cigarette smoke, contamination also occurs through ingestion of contaminated food and water. 

 The International Agency for Research on Cancer and the National Toxicology Program has labeled benzene as a definite human carcinogen. Various studies point to a correlation between benzene exposure and breast cancer risk. Laboratory studies on mice have shown that a high level of benzene exposure can lead to mammary cancer [66]. Mice exposed to benzene displayed frequent mutations of genes that are responsible for suppressing the development of tumors [67]. In one epidemiological study that looked at relevant occupations among female Chinese workers, the occupations in which elevated risks for breast cancer were found to include scientific research workers, medical and public health workers, and electrical, and electronic engineers, as well as teachers, librarians, and accountants. Benzene exposure was associated with an elevated risk of breast cancer among exposed professionals [68]. Results from other recent studies support these conclusions, including a study examining occupational exposures among enlisted women in the US Army [69] and women in various professions in Israel [70]. Another study of a fairly small sample of women for whom researchers had benzene exposure data from their work at a shoe factory in Florence, Italy, also supported a relationship between exposure to benzene and later development of breast cancer [71].

### 5.2. Methylene Chloride

This is a highly volatile compound used in various consumer products like fabric cleaners, paint strippers, wood sealant and stains, spray paint, adhesives, furniture and shoe polish, and art supplies among other. Exposure occurs mainly during production and industrial use of methylene chloride and dichloromethane alongside the environmental exposure resulting from the use of the consumer products [72]. High levels of exposure are associated with benign mammary tumors in animal experiments involving rats. The study established increased incidences of fibroadenomas of the mammary gland in female rats who inhaled methylene chloride [65].

Once inhaled or absorbed into the body, methylene chloride is converted to carbon monoxide which interferes with oxygen delivery, hence causing angina (chest pain) and making other heart symptoms worse in people with heart disease. People with lung conditions, smokers, and overweight or pregnant people may also be more sensitive to methylene chloride. When inhaled or absorbed through the skin, methylene chloride can reach the developing fetus in pregnant women through the placenta and also enter breast milk. Methylene chloride affects the nervous system (brain) and can cause headaches, dizziness, nausea, clumsiness, drowsiness, and other effects like those of being drunk. When exposed to high levels of methylene chloride frequently over months or years, the nervous system effects can be long-lasting and possibly permanent. Methylene chloride causes cancer in laboratory animals and potentially can cause cancer in humans. 

### 5.3. Ethylene Oxide

Ethylene oxide is used to sterilize medical equipment and other products like clothing, cosmetics, and beekeeping equipment. It is also found in tobacco smoke, exhaust gases, and some foods and spices. Occupational exposure is the greatest among people who work directly with the compound. Ethylene oxide is a known human carcinogen. One study found a twofold increased risk of breast cancer (standardized morbidity ratio) among women who worked in an industry with documented exposure [41]. In a cohort study of 7576 women employed for at least one year and exposed for an average 10.7 years while working in commercial sterilization facilities, breast cancer incidence (*n* = 319) was ascertained via interview, death certificates, cancer registries, and medical records [73]. Despite contradictory data in the recent literature, other reports support the finding that exposure to ethylene oxide is associated with increased risk for breast cancer in women [74]. Studies in which human breast cells grown in vitro were exposed to low doses of ethylene oxide demonstrated that the chemical exposure resulted in a significant increase in damage to the cells' DNA [74].

### 5.4. Styrene

Styrene is used in manufacturing and is a byproduct of polystyrene (plastic). It is also used in the synthetic rubber industry. Many building materials, home maintenance products, and several consumer products like carpets, paints, adhesives hobby, and craft supplies contain styrene [13, 65]. General exposure occurs by inhalation of contaminated air and tobacco smoke as well as ingesting contaminated food and water. 

Styrene has been classified as a possible carcinogen by the National Toxicology Program [75]. Several animal studies have linked styrene with increased mammary tumors [65]. Results from these studies may not be necessarily generalized to humans because, for a given mode of absorption, major differences exist between species in uptake, distribution, and metabolism [61]. For example, higher quantities of styrene oxide are produced by mice than rats or humans after exposure to styrene. Humans are also found to have the highest capacity to metabolize styrene oxide than mice or rats [76]. At the same time, most animal studies are based on exposure to a single organic solvent. Human exposures on the contrary are due to mixture exposures and other possible carcinogens and thus interactions between different solvents may inhibit or potentiate known effects of individual solvents [11].

### 5.5. Tetrachloroethylene

Tetrachloroethylene (PCE), also known as perchloroethylene or perc, is an important occupational chemical used in metal degreasing and dry cleaning and a prevalent drinking water contaminant. Human exposure is mainly by inhalation of vapors, by direct contact with the skin, or by ingesting contaminated water or food. The highest exposures trends to tetrachlorethylene occur in the workplace, especially among dry cleaning and degreasing workers. Workers in some other industries may also be exposed to tetrachlorethylene. 

Tetrachloroethylene has been categorized as carcinogenic by IARC. There are hypothesized mode(s) of action only for rat kidney tumors and mouse liver tumors. For the former, the hypothesized modes of action include mutagenicity, peroxisome proliferation, *α*_2u_-globulin nephropathy, and cytotoxicity not associated with *α*_2u_-globulin accumulation. For mouse liver tumors, the hypothesized mode(s) of action include mutagenicity, epigenetic effects (especially DNA hypomethylation), oxidative stress, and receptor activation (focusing on a hypothesized PPAR*α* activation mode of action). However, the available evidence is insufficient to support the conclusion that either rat kidney or mouse liver tumors are mediated solely by one of these hypothesized modes of action. Epidemiological studies found elevated levels of breast cancer in working women in drycleaners [16]. One population-based case control study of women who were accidentally exposed to Perc leaching from improperly prepared water pipes found an elevated risk of breast cancer associated with exposure [77].

### 5.6. Other Organic Compounds

There are several other organic solvents including industrial chemicals that were believed to be associated with breast cancer. Some of these include carbon tetrachloride, urethane, toluene diisocyanate mixtures, isoprene nonylphenols, and 1, 3-butadiene among others. Most of these chemicals enter the body by inhalation or ingestion in contaminated food and drinks. The Occupational Safety and Health Administration (OSHA) produces cancer risk estimates of various organic chemicals to determine permissible exposure limits in workplaces. Whereas these risk estimates are not specific to breast cancer, the overall risks are as high as one percent indicating that workers can face a substantial danger when exposed. 

## 6. Conclusion 

Carcinogenic compounds including metals and organic solvents play a major role in the multistep processes that lead to breast cancer, including initiation, promotion, progression, and metastasis. A combination of biological processes and physiochemical effects of each carcinogen in conjunction with other factors including genetics, age, and body size can contribute to development of the breast cancer. In spite of the wide range of physiochemical properties of metal compounds, some common mechanism of metal carcinogenesis emerges in general. These include induction of oxidative stress, inhibition of DNA repair, activation of mitogenic signaling, and epigenetic modification of gene expression. On the other hand, organic solvents can cause breast cancer through various mechanism including intact solvent molecules reaching the breast epithelium where they are bioactivated into electrophilic metabolites that cannot be detoxified due to reduced activity of the necessary enzymes within the breast cells, some of electrophilic metabolites produced in other organs like liver or kidney are transported to the breast accumulating in the milk ducts and causing toxic effects, and finally estrogenic effects, of endogenous and exogenous substances. Even though little epidemiological evidence exists especially on the effects of organic solvents, the fact that these compounds have been categorized as carcinogenic with some evidence in experimental animals is enough to ensure appropriate regulation is in place to protect environmental contamination of both carcinogenic metal and organic solvents. Occupational exposure must be minimized since this is the route through which the majority are affected. Human activity leading to environmental pollution must be monitored by authorized organs to ensure that the populations are protected from the toxicity of these potentially dangerous compounds. 

## Figures and Tables

**Table 1:** Organic solvents detected in human milk.

Organic solvent	References
Acetaldehyde	a
Benzaldehyde	a
Benzene	a
Carbon disulfide	a–c
Carbon tetrachloride	c
Chlorobenzene	a
Chloroethane (ethyl chloride)	a
Chloromethane	a
Chloropentane	a
Crotonaldehyde	a
Cyclohexane	a
Cyclopentane	a
Dichlorobenzene	a
1-2-Dichloroethane	a
Dichloroethylene	c
Ethyl alcohol	a
Ethylbenzene	b
Ethyl methyl ketone	a
*Β*-Hexachlorocyclohexane	a
Methyl alcohol (methanol)	a
Methyl amyl ketone (2-heptanone)	a
Methyl ethyl ketone	a
Methyl isobutyl ketone	a
Methyl propyl ketone	a
Methylene chloride	a
Styrene	a, c
Tetrachloroethene	a, b
Toluene	a
1,1,1-Trichloroethene	a
Trichloroethylene	
Trichloromethane (chloroform)	a
Xylenes	a

a: Pellizzari et al., 1982 [23]; b: Wolff, 1983 [24]; c: Jensen, 1991 [25].
